# Live-virus exposure of vaccine-protected macaques alters the anti-HIV-1 antibody repertoire in the absence of viremia

**DOI:** 10.1186/1742-4690-10-63

**Published:** 2013-06-21

**Authors:** Barbara C Bachler, Michael Humbert, Samir K Lakhashe, Robert A Rasmussen, Ruth M Ruprecht

**Affiliations:** 1 Department of Cancer Immunology and AIDS, Dana-Farber Cancer Institute, Boston, MA 02215, USA; 2 VetCore Facility for Research, University of Veterinary Medicine, 1210, Vienna, Austria; 3 Harvard Medical School, Boston, MA 02215, USA

**Keywords:** Macaque model, Recombinant protein vaccine, Heterologous R5 SHIV clade C challenge, Complete protection, Subtractive peptide phage display, V3 crown, Boosting of vaccine-induced Ab titers after multiple challenges, Neo-antigen reactivity

## Abstract

**Background:**

We addressed the question whether live-virus challenges could alter vaccine-induced antibody (Ab) responses in vaccinated rhesus macaques (RMs) that completely resisted repeated exposures to R5-tropic simian-human immunodeficiency viruses encoding heterologous HIV clade C envelopes (SHIV-Cs).

**Results:**

We examined the Ab responses in aviremic RMs that had been immunized with a multi-component protein vaccine (multimeric HIV-1 gp160, HIV-1 Tat and SIV Gag-Pol particles) and compared anti-Env plasma Ab titers before and after repeated live-virus exposures. Although no viremia was ever detected in these animals, they showed significant increases in anti-gp140 Ab titers after they had encountered live SHIVs. When we investigated the dynamics of anti-Env Ab titers during the immunization and challenge phases further, we detected the expected, vaccine-induced increases of Ab responses about two weeks after the last protein immunization. Remarkably, these titers kept rising during the repeated virus challenges, although no viremia resulted. In contrast, in vaccinated RMs that were not exposed to virus, anti-gp140 Ab titers declined after the peak seen two weeks after the last immunization. These data suggest boosting of pre-existing, vaccine-induced Ab responses as a consequence of repeated live-virus exposures. Next, we screened polyclonal plasma samples from two of the completely protected vaccinees by peptide phage display and designed a strategy that selects for recombinant phages recognized only by Abs present *after* – but not before – any SHIV challenge. With this “subtractive biopanning” approach, we isolated V3 mimotopes that were only recognized after the animals had been exposed to live virus. By detailed epitope mapping of such anti-V3 Ab responses, we showed that the challenges not only boosted pre-existing binding and neutralizing Ab titers, but also induced Abs targeting neo-antigens presented by the heterologous challenge virus.

**Conclusions:**

Anti-Env Ab responses induced by recombinant protein vaccination were altered by the multiple, live SHIV challenges in vaccinees that had no detectable viral loads. These data may have implications for the interpretation of “vaccine only” responses in clinical vaccine trials.

## Background

According to UNAIDS/WHO, more than 2 million people are newly infected with HIV-1 each year [1]. The design of an effective vaccine is important to control the global expansion of the AIDS pandemic [2]. In the context of vaccine efficacy in humans, only the RV144 trial showed promising results so far [3] and recent follow-up studies identified vaccine-induced correlates of protection [4,5]. Additionally, biologically relevant non-human primate (NHP) models are used in HIV-1/AIDS research to gain information about vaccine-induced immunity [6,7]. In contrast to clinical studies in humans, NHPs can be deliberately challenged with well characterized virus inocula. This allows a subsequent detailed analysis of virus-specific consequences on pre-existing (vaccine-induced) immune responses.

Previously, we described different immunization/challenge studies in rhesus macaques (RMs) that were vaccinated with recombinant protein immunogens (multimeric HIV-1 clade C (HIV-C) gp160, HIV-1 Tat and SIV Gag-Pol particles). Upon repeated challenge with the heterologous R5-tropic simian-human immunodeficiency viruses encoding HIV-1 clade C envelopes (SHIV-Cs), all controls became infected and developed high peak viremia, whereas some vaccinees remained aviremic throughout ([8-12] and unpublished). Here, we focused on the antibody (Ab) responses in vaccinees that had resisted all mucosal challenges completely and asked two questions: i) is there a *quantitative* difference in the anti-Env Ab titers after versus before live-virus exposures in animals without detectable viremia? And ii) is there a *qualitative* difference in the Ab responses after versus before live-virus exposures in the same animals due to newly induced Abs targeting neo-antigens that were presented by the heterologous challenge virus? To address these issues, we decided to dissect the Ab responses in completely protected RMs further and to take an imprint of the Ab paratopes after virus challenges using recombinant phage libraries encoding random peptides.

## Results

### Dynamics of anti-Env Ab responses in vaccinated RMs

**Figure 1:**
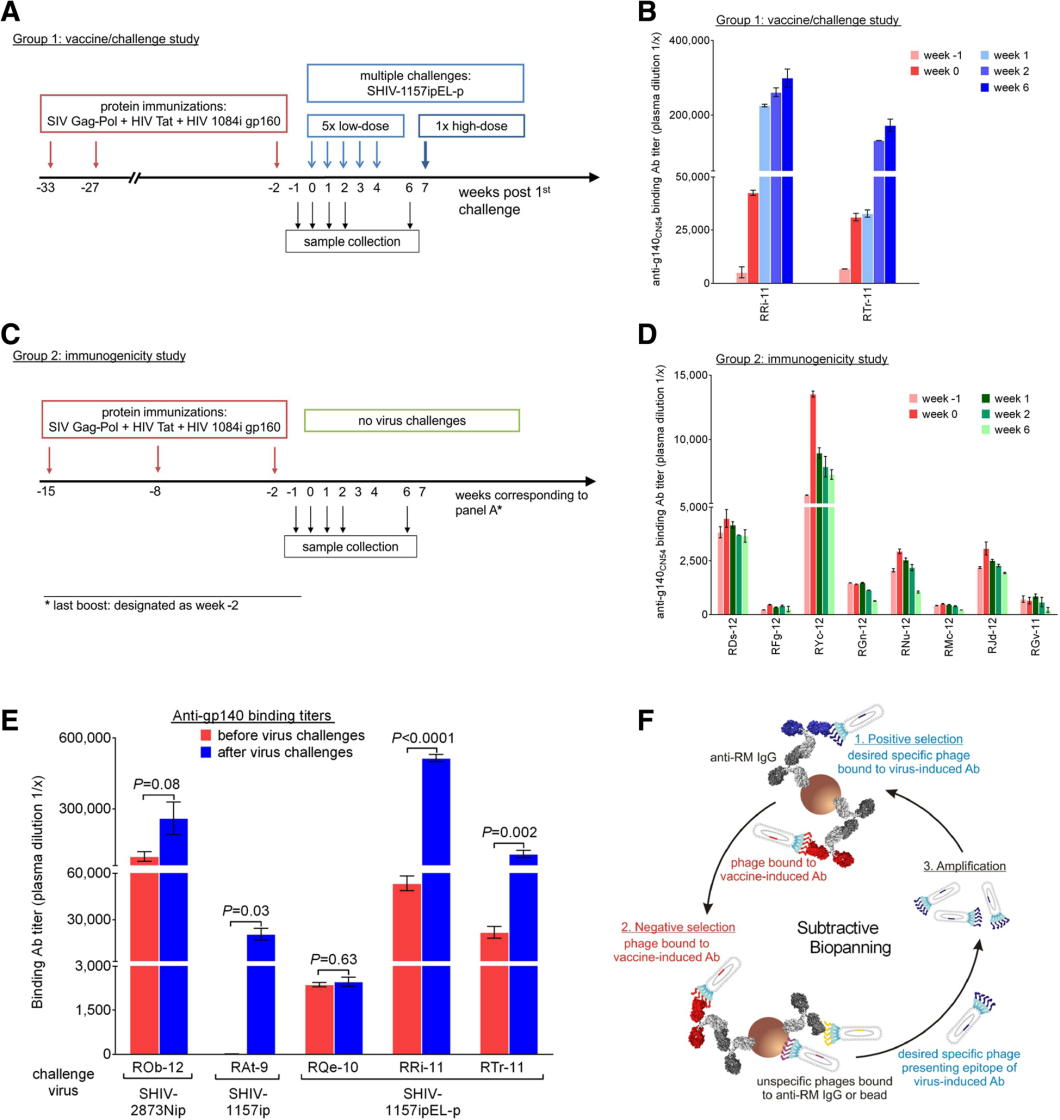
**Anti-Env Ab responses in vaccinated RMs before and after virus challenge. A.** Time line of vaccine/challenge study [12]. In red, immunization phase; in blue, challenge phase. Plasma samples were collected at weeks −1, 0, 1, 2 and 6. **B.** Quantitative ELISA to determine Env-specific Ab titers in two vaccine-protected RMs. In red, time points post 3^rd^ immunization and pre-challenge; in blue, time points post challenge. **C.** Time line of immunogenicity study (unpublished). The last protein immunization was designated as week −2, so that the subsequent weeks would correspond to the study in (**A**). **D.** Quantitative ELISA to determine Env-specific Ab titers in eight vaccinated, but not challenged RMs. In red, 1 or 2 weeks post 3^rd^ immunization; in green, subsequent time points. **E.** The gp140_CN54_-specific Ab titers were compared before and after live-virus challenges in five completely protected vaccinees. The challenge viruses used are indicated. Height of each bar, average titer from three independent assays; error bars, standard error of the mean (SEM). *P* values are shown (*P* < 0.05 was considered significant). **F.** Subtractive biopanning. Three rounds of selection were performed to identify Ab epitopes linked to live-virus exposure. Each round of selection consists of (1) positive selection, (2) negative selection and (3) amplification of the selected phages. Light gray, the Fc portion of all Abs. Dark gray, Fab portion of anti-RM IgG immobilized onto paramagnetic beads via the Fc. Positive selection used week 7 plasma from a protected animal. In dark blue, Fab portions of the live-virus induced Abs and the corresponding phages. Positively selected recombinant phages were counter-selected with plasma from the same vaccinee but collected at week 0 (containing vaccine-induced Abs only). In red, Fab portions of negative selector Abs and the corresponding bound phages. Purple or yellow phages, unspecific phages bound to anti-RM Ab or beads, respectively.

To test whether live-virus exposures could induce *quantitative* changes in the vaccine-induced Ab responses in RMs where the virus failed to cause any detectable viremia, we first investigated Env-specific plasma Ab titers at time points *before* and *after* virus challenge. We examined plasma samples of two vaccine-protected RMs, RRi-11 and RTr-11, that had been enrolled in the same vaccine/challenge study [12] and challenged multiple times with the R5 clade C SHIV-1157ipEL-p [13] (Group 1 in Figure 1A, B and Table 1). Monkey RRi-11 fulfilled all criteria for sterilizing immunity, whereas RTr-11 showed anamnestic cellular immune responses compatible with cryptic infection ([12] and Table 1). As a control, we also investigated anti-Env binding Ab titers in eight animals that had been part of an unpublished immunogenicity study (Group 2, Figure 1C, D). Importantly, these animals were immunized similarly as the monkeys from Group 1, including the same adjuvant (incomplete Freund’s adjuvant, IFA). To allow a direct comparison of both groups, we adjusted the time points for Group 2 and designated the time of last protein immunization as week −2. For both groups, we tested plasma collected at weeks −1 and 0 and up to 8 weeks post last protein immunization (weeks 1, 2 and 6). Most animals showed an increase of anti-gp140 Ab titers between week −1 (light red) and week 0 (dark red) (Figure 1B, D), which reflects the expected boosting of Ab responses during the two weeks after the last protein immunization (week −2). Yet, when we examined the anti-gp140 binding Ab responses at later time points, only the two vaccinees exposed to live virus showed a continuing increase of anti-gp140 Ab titers (Group 1, blue bars, Figure 1B). In contrast, the Ab levels in Group 2 controls peaked at the two weeks post last immunization and declined during the time window that corresponds to the virus challenge in Group 1 (green bars, Figure 1D). Taken together, we conclude that the third protein immunization led to a boosting of anti-gp140 Ab responses, which reached a peak within two weeks (week 0). Importantly, these Env-specific Ab titers continued to increase only in the RMs exposed repeatedly to live virus, although no viremia was ever detected. These data suggest a boosting of anti-Env Abs by virus challenges that did not result in systemic infection.

**Table 1:** Immune status of vaccinees after all live-virus challenges

**Level of protection**	**Animal name**	**Virological outcome**	**Interpretation**	**Immunogens**	**Challenge viruses (challenge route)**	**Enrolled in study**
**Complete**	**ROb-12**	Aviremic	Cryptic infection	HIV-C gp160 and gp145 + SIV Gag overlapping synthetic peptides (OSP)+ HIV Tat OSP+ SIV Nef OSP	Multiple low-doses with SHIV-2873Nip (intrarectal) [14]	unpublished
	**RAt-9**	Aviremic	Cryptic infection	HIV-C gp160 + SIV Gag-Pol particles + HIV Tat	Single low-dose with SHIV-1157ip (oral) and single high-dose with SHIV-1157ipd3N4 (intrarectal) [15]	[8-10]
	**RQe-10**	Aviremic	Sterilizing immunity	*Listeria monocytogenes* expressing SIV *gag* + Ad5hr encoding SIV *gag* + HIV-C gp160 + HIV Tat	Multiple low-doses with SHIV-1157ipEL-p (intrarectal) [13]	[11]
	**RRi-11**	Aviremic	Sterilizing immunity	HIV-C gp160 + SIV Gag-Pol particles + HIV Tat	Multiple low-doses and one high-dose with SHIV-1157ipEL-p (intrarectal) [13]	[12]
	**RTr-11**	Aviremic	Cryptic infection			
**Partial**	**RBr-11**	Lower peak viremia	Chronic systemic infection			
	**RGe-11**	Aviremic during low-dose challenges	2 low-level blips (<10^4^ copies/ml)			
**None**	**RDo-11**	No protection	Chronic systemic infection			

Next, we extended our analysis and examined the anti-Env Ab binding titers in three additional vaccinees that were also completely protected but had been enrolled in different vaccine/challenge studies ([8-11] and unpublished). The immunogens and challenge viruses used are listed in Table 1. Although the multigenic immunogen composition had varied among the studies, all of the vaccinees had received the same multimeric HIV-C gp160. When we compared the vaccine-induced anti-gp140 Ab titers (Methods) with the Ab responses against the same protein but measured *after* virus exposures, we observed a statistically significant increase of anti-Env specific Abs not only for the two RMs mentioned, RRi-11 and RTr-11 (*P* < 0.0001 and *P* = 0.002, respectively), but also for RM RAt-9 (*P* = 0.03), which had been challenged with a different virus (Figure 1E). Also, the protected RM ROb-12, which had resisted five low-dose challenges with the tier 2 SHIV-2873Nip (unpublished data), showed a trend towards higher Env-specific plasma Ab titers after the live-virus encounters (*P* = 0.08). In the fifth aviremic vaccinee, RQe-10, the anti-Env binding Ab titers did not change.

Of note, we have also recently examined anti-Tat Ab responses for animals RAt-9, RQe-10, RRi-11 and RTr-11 before and after live-virus exposures [16]. We observed a similar boosting of Ab levels in the same animals (again no changes for RQe-10). Combined, these data show measurable boosting of at least two different HIV-1 targets, Env and Tat, after live SHIV-C exposures – although no viral loads were ever detected in these animals throughout the time course of observation that ranged from one to six years.

### Probing the virus-specific Abs: Subtractive biopanning

Based on these *quantitative* changes, we addressed the question whether the virus challenge had also induced a *qualitative* change in the Abs responses. In other words, we sought to examine whether there were Abs with new specificities present only *after* – but not before – any exposure to live virus. To probe the paratopes of these new Abs, we further dissected the Ab repertoire in the two aviremic animals mentioned above (RRi-11 and RTr-11), using recombinant phage libraries encoding random peptides. To identify Ab responses only present after exposure to the challenge virus (in this case SHIV-1157ipEL-p [12]), we devised a novel selection strategy based upon random peptide phage display and termed “subtractive biopanning”: Positive selection used plasma from a completely protected RM after all low-dose exposures (week 7). This was followed by negative counter-selection with plasma from the same animal collected two weeks after the last protein boost, corresponding to just before the first virus challenge (week 0) (Figure 1F). We hypothesized that removing phages recognized by vaccine-induced Abs would enrich for phages binding to Abs generated during the repeated mucosal virus challenges. Three rounds of alternating positive/negative selection were performed. Peptide inserts of selected recombinant phages (phagotopes) were sequenced and aligned with the Env sequence of the immunogen (HIV1084i gp160) or the challenge strain (SHIV-1157ipEL-p) to search for mimicry (mimotopes).

**Figure 2:**
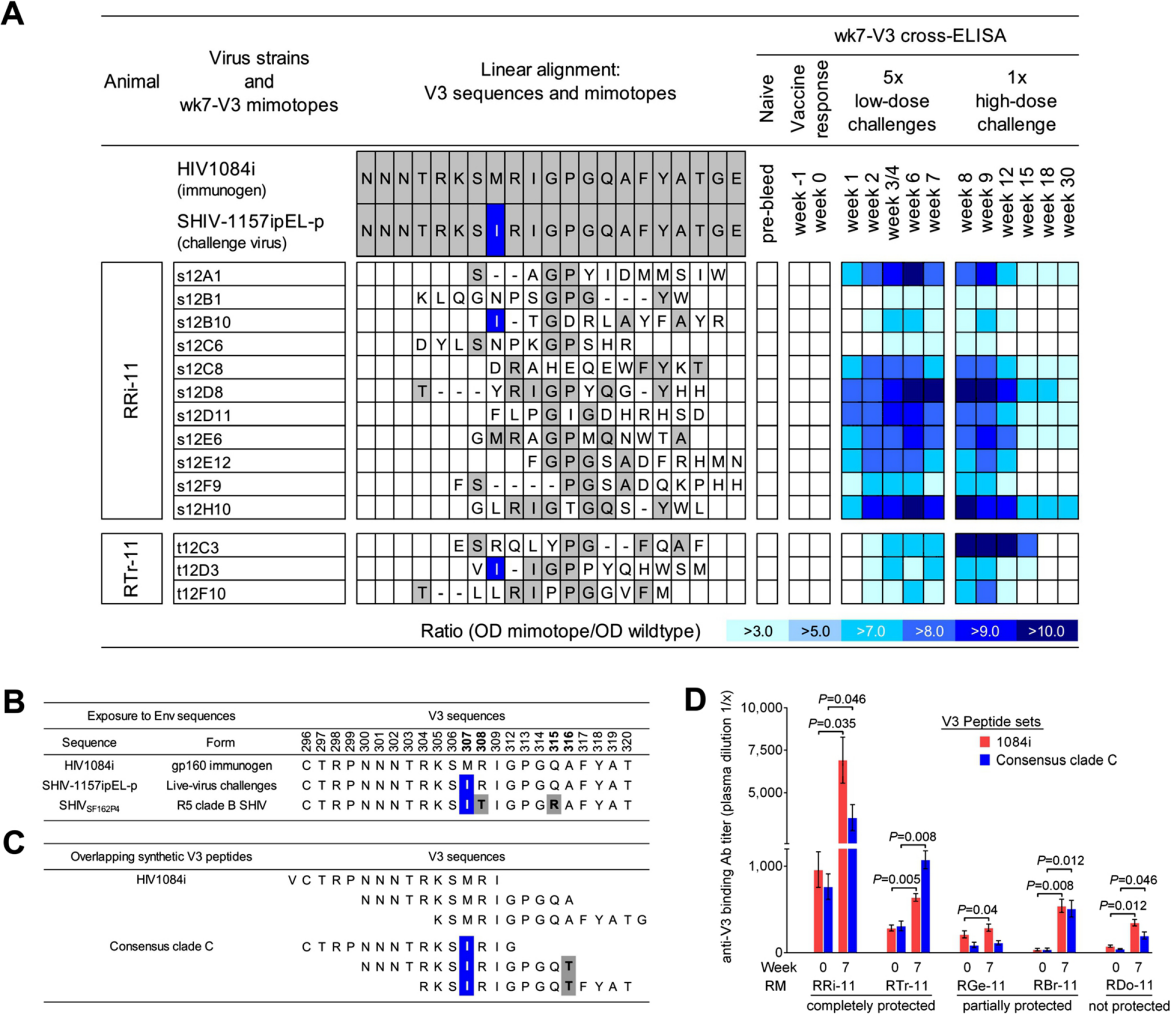
**Mapping of anti-V3 binding Abs. A.** Sequences of recombinant phages were assigned to V3 crown of HIV1084i or SHIV-1157ipEL-p. Gray shading, linear homologies; blue shading/white letters, amino acid difference between the two HIV-C envelopes (M307I). V3 mimotopes isolated using week 7 plasma (wk7-V3 mimotopes) were tested for plasma Ab binding using 14 time points of RRi-11 and RTr-11. Negative control, pre-immune plasma; weeks −1 and 0, vaccine-induced Ab responses; weeks 1–7, also include Ab responses induced during low-dose virus challenges; weeks 8–30, Ab responses after all challenges. Data illustrate results from two independent assays. Binding patterns are shown in form of a heat-map. OD signals 3x higher than signals detected with the wildtype phage control were considered positive. **B.** V3 amino-acid sequences of HIV1084i, SHIV-1157ipEL-p and SHIV_SF162P4_. The two HIV clade C envelopes of SHIV-1157ipEL-p and HIV1084i differ in only one residue in the V3 crown (M307I; HXB2 numbering scheme [17]; highlighted in blue). SHIV_SF162P4_ shares the same gp120-sequence as HIV_SF162_[18] and has three mutations compared with the 1084i immunogen (M307I (blue), and R308P and Q315K (gray)). **C.** Sequences of synthetic peptide sets used for plasma Ab titration and peptide absorption analysis. Peptides corresponding to consensus clade C sequence differed in two amino-acid residues compared with immunogen-related peptides (HIV1084i): M307I (blue) and A316T (gray). **D.** Plasma samples of five vaccinees [12] were assessed for binding Ab specificities at weeks 0 and 7 using two different V3 peptide sets (red bars, 1084i; blue bars, consensus clade C). Height of each bar, average titer calculated from three independent assays; error bars, standard error of the mean (SEM). Statistically significant differences between weeks 0 and 7 are indicated (if *P* < 0.05).

Depending on the positive selector used, different motifs were identified (data not shown). However, one recurring motif shared by RRi-11 and RTr-11 could be assigned to the V3 crown (linear alignment, Figure 2A), and the corresponding mimotopes were termed wk7-V3 mimotopes. To verify that the latter represented epitopes of Abs induced by the virus challenges rather than Abs induced by the gp160 1084i immunogen, we cross-tested 14 different plasma samples from each aviremic animal for specific binding by phage ELISA. The results are presented in the form of a heat-map (Figure 2A). None of the wk7-V3 mimotopes were recognized by plasma Abs present before live-virus encounters (naïve, week −1 or week 0 samples). However, once the vaccinees were exposed to SHIV-1157ipEL-p for the first time (week 1), 8 out of 14 wk7-V3 mimotopes were specifically recognized by plasma Abs, with increasing binding reactivities at week 2. Interestingly, these responses were strongly boosted by the single high-dose rechallenge with the same SHIV-C (Figure 2A). Overall, these data confirmed that our subtractive selection strategy had indeed enriched for mimotopes only recognized by Abs present *after* live SHIV-C exposures.

### Mapping of vaccine- versus virus-specific V3 Ab epitopes

The vaccinees had encountered two different HIV-C Env sequences, HIV1084i as an immunogen and SHIV-1157ipEL-p as challenge virus. HIV1084i and SHIV-1157ipEL-p Envs are distinct and only share 78% amino acid (AA) homology [12]. Importantly, they differ in the V3 crown at position 307 (methionine to isoleucine switch; M307I) (Figure 2B), which affects one of three V3 core residues recently described as essential for the recognition of broadly neutralizing antibodies (bnAbs) [19]. For epitope mapping, we used two different peptide sets corresponding to the V3 crown (Figure 2C): one was identical to the HIV1084i immunogen sequence, while the other set represented a consensus clade C sequence and contained M307I as in the heterologous challenge virus, SHIV-1157ipEL-p. Of note, the consensus C V3 peptide set shows another AA switch (A316T) in the V3 circlet. However, this area is more variable and therefore less likely targeted by bnAbs [19].

Using the linear V3 peptides (Figure 2C), we performed binding ELISAs to confirm that the low-dose SHIV-1157ipEL-p exposures had altered Ab titers. Towards this end, we analyzed plasma samples collected at weeks 0 and 7 in the two vaccine-protected RMs, RRi-11 and RTr-11 (Figure 2D). As a comparison, we also examined three vaccinees that had been part of the same study ([12], Table 1 and time line in Figure 1A) but had detectable viral RNA: RGe-11 exhibited two transient, low-level blips (<10^4^ viral RNA copies/ml) but lymph node biopsies were virus negative. RBr-11 developed chronic systemic infection but had lower peak viremia compared with the controls and RDo-11 was chronically infected without any signs of protection (Table 1).

Remarkably, the aviremic animals RRi-11 and RTr-11 showed significantly higher immunogen-related V3-specific ELISA titers after the fifth virus challenge compared with the titers before the first live-virus encounter (Figure 2D, red bars; *P* = 0.035 and *P* = 0.005, respectively). As expected, the three other vaccinees that had detectable viral loads (RGe-11, RBr-11 and RDo-11) also showed a statistically significant boosting using the immunogen-related 1084i V3 peptides (red bars, *P* = 0.04, *P* = 0.008 and *P* = 0.012, respectively). Thus, the 5x low-dose challenges with SHIV-1157ipEL-p boosted the vaccine-induced anti-V3 binding Ab titers in all five animals tested.

When we used the consensus C V3 peptides (blue bars), which contain the critical mutation (M307I) that is present in the challenge virus but not in the immunogen, we detected an increase of anti-V3 binding Abs in the two aviremic vaccinees, RRi-11 (*P* = 0.046) and RTr-11 (*P* = 0.008), as well as in RBr-11 (*P* = 0.012) and RDo-11 (*P* = 0.046). The anti-consensus C V3 Ab titers did not change for RM RGe-11 when samples from weeks 7 and 0 were compared (Figure 2D). Taken together, our data indicate that the five SHIV-1157ipEL-p challenges significantly boosted the pre-existing anti-V3 binding Ab responses. Importantly, this was also observed in two protected RMs, although no viremia was detected throughout.

### Boosting of vaccine-induced anti-V3 nAbs in the absence of viremia

**Figure 3:**
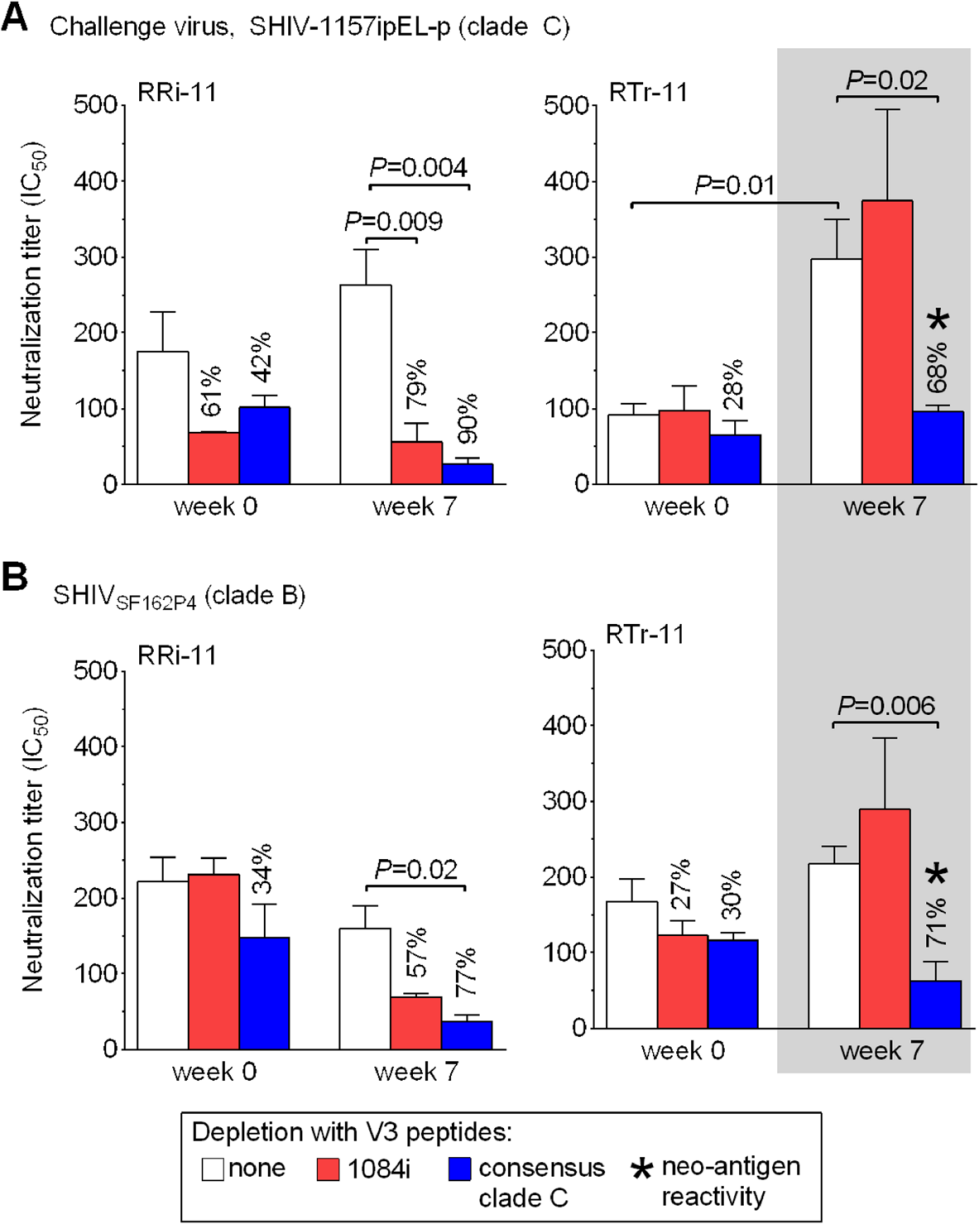
**Induction and boosting of cross-neutralizing anti-V3 Abs in the absence of viremia.** Plasma samples (weeks 0 and 7) were tested for neutralizing activity against the challenge virus, SHIV-1157ipEL-p (**A**) and the heterologous clade B SHIV_SF162P4_ (**B**) by TZM-bl assay [20]. Neutralization titers are expressed as the reciprocal plasma dilution inhibiting 50% of virus infection (IC_50_). Each bar represents the average titer of at least two independent assays using triplicates of each sample and error bars show the SEM. Plasma nAb titers were compared between weeks 0 (before live-virus challenges) and 7 (after 5x multiple low-dose challenges). Plasma samples were incubated with either medium (plasma only, white bars) or one of the two peptide sets (red bars, 1084i V3; blue bars, consensus clade C V3) and IC_50_ values were calculated. In case of a decreased IC_50_ (depletion of neutralizing activity), percentages illustrate the degree of inhibition reached with each peptide set. *P*-values are shown (significance after Bonferroni correction, *P* < 0.025). Gray box and asterisks indicate neoantigen reactivity.

To test whether the multiple SHIV-1157ipEL-p challenges had not only altered Ab binding activity but also the vaccine-induced nAb activity, we examined plasma samples of weeks 0 and 7 for nAb titers. Although no viremia was ever detected in animals RRi-11 and RTr-11, their 50% inhibitory concentration (IC_50_) against the challenge virus increased after the 5× virus encounters (Figure 3A; white bars, IC_50_ of weeks 0 vs. 7). For RRi-11, the IC_50_ was insignificantly 1.5-fold higher. For RTr-11, we detected a statistically significant 3.3-fold increase (*P* = 0.01) at week 7. Clearly, live-virus exposures boosted pre-existing nAb responses in this aviremic vaccinee.

Next, we examined whether the increased neutralization titer noticed after live SHIV-C exposures could be linked to anti-V3 nAb responses. Thus, we absorbed the neutralizing activity by incubating plasma in the presence/absence of the different V3 peptide sets shown in Figure 2C. Absorption with the immunogen-related 1084i V3 peptides reduced the neutralizing activity of the week 7 plasma by 79% in RRi-11 (*P* = 0.009) but not at all in RTr-11 (Figure 3A; red bars). Importantly, absorption with the consensus C V3 peptides significantly lowered the neutralizing activity *after* but not *before* live-virus exposure in both aviremic animals (blue bars; *P* = 0.004 and 0.02, respectively). Neither the partially protected nor the non-protected monkeys showed changes in nAb titers or V3 reactivity (RGe-11, RBr-11, and RDo11, data not shown). Taken together, the peptide absorption data imply that nAbs with new target specificity had developed between weeks 0 and 7, especially in animal RTr-11 (neo-antigen reactivity; gray box and asterisk in Figure 3A).

### Induction of new cross-neutralizing anti-V3 antibodies in the absence of viremia

We reasoned that 5× live-virus exposures might have induced anti-V3 nAbs that cross-neutralize a heterologous, non-clade C virus and tested plasma samples of weeks 0 and 7 for neutralizing activity against the clade B SHIV_SF162P4_[21]. We used the peptide absorption described above to link neutralization to anti-V3 Abs and noted three AA changes in the V3 crown of SHIV_SF162P4_ compared with the HIV1084i immunogen (M307I, R308T and Q315R; see Figure 2B). Overall, the 5× SHIV-1157ipEL-p challenges did not increase neutralizing activity against SHIV_SF162P4_ in the two aviremic RMs (Figure 3B; white bars, IC_50_ of weeks 0 vs. 7). We also saw no statistically significant differences in IC_50_ levels at week 7 when using the immunogen-related 1084i V3 peptides (red bars). In contrast, peptide absorption showed highly significant drops in IC_50_ values after incubation with the consensus C V3 peptides (blue bars, *P* = 0.02 for RRi-11 and *P* = 0.006 for RTr-11). These data are consistent with an expansion of target specificity in both protected animals (neo-antigen reactivity for RTr-11; gray box and asterisk in Figure 3B) at week 7. No significant changes were seen for the partially protected animal RGe-11 (data not shown).

Overall, we conclude that the 5× live-virus exposures: i) boosted vaccine-induced anti-1084i V3 binding Ab titers, ii) induced anti-V3 nAb responses against the challenge virus SHIV-1157ipEL-p, and iii) changed the V3 loop specificity of nAbs against heterologous SHIV_SF162P4_ in two animals without any signs of viremia.

## Discussion

Here we describe: i) a *quantitative* change in the Env-specific plasma Ab titers in four out of five persistently aviremic vaccinees, ii) subtractive biopanning with recombinant phages encoding random peptides as a new tool to dissect *qualitative* (virus-induced) changes in the humoral immune responses of vaccine-protected animals, iii) an increase of vaccine-induced anti-V3 binding Abs and induction of novel, cross-neutralizing anti-V3 Abs due to live SHIV-C encounters in animals that had remained virus-free.

Different immunization studies performed by our group yielded a cohort of five vaccinees that resisted multiple live SHIV-C exposures completely. Here, we dissected the Env-specific Ab responses in these monkeys. More specifically, we investigated the consequences of live-virus challenges on the strength and specificity of vaccine-induced Ab responses in the absence of any detectable viral load. Unexpectedly, we detected higher Env-specific binding Ab titers in four out of five aviremic RMs after they had been exposed to live SHIV-C. The boosting of Ab responses was independent of the challenge route. Similar effects were observed after oral (for animal RAt-9 [8-10]) and intrarectal challenges (for all the other monkeys tested).

To verify that the increased anti-Env Ab titers were a direct consequence of the virus challenges, we compared the dynamics of anti-gp140 Ab responses in vaccinated RMs with or without subsequent exposures to live virus. As expected, we detected a vaccine-induced boost of Ab responses about two weeks after the last protein immunization in both groups of animals. Remarkably, these titers kept increasing during the repeated virus challenges although no viremia ensued. In contrast, immunization without subsequent virus challenges resulted in the expected increase in anti-gp140 Ab levels that peaked at week 2 post-immunization and continuously declined thereafter. These data confirmed a live-virus induced increase of pre-existing, vaccine-induced Ab responses.

We sought to examine the Ab repertoire in two of the protected animals further and determined whether the live-virus challenges only boosted the pre-existing, vaccine-induced responses or also induced new Abs against neo-antigens represented by the Env proteins on the challenge virus particles. Phage display [22] has been used to analyze humoral immune responses in the context of chronic infections with immunodeficiency viruses [23-27]. Here, we designed a novel subtractive biopanning strategy to probe the paratopes of virus-induced Abs in vaccine-protected animals. We hypothesized that depleting phages recognized by Abs present after immunization but before any virus exposures would select for phages recognized by live virus-induced Abs only. We performed subtractive biopannings using polyclonal plasma samples from completely protected vaccinees and analyzed the selected phagotopes for similarities to Env sequences. This approach allowed us to identify the V3 crown as an epitope specifically recognized by week 7 but not week 0 Abs and shared by the two aviremic vaccinees, although subtractive biopanning revealed other Env regions for these vaccinees individually (data not shown).

Recently, the V3 crown and its conserved structural elements that are involved in co-receptor binding were shown to be important targets for bnAbs, including mAbs 447/52-D, 2219, 3074, 33B2, 33C6 [19,28-30] and HGN194 [31]; the latter also provided complete cross-clade protection against SHIV-C acquisition *in vivo*[32]. Together with the novel bnAbs, PG9 and PG16, targeting quaternary epitopes formed by the V2 and V3 loops in trimeric Env [33], the V3 loop is now considered again as target for vaccine development (reviewed in [28]).

By phage ELISA, we confirmed that the V3 mimotopes isolated by subtractive biopanning were only recognized *after* but not before the first virus challenge. Importantly, these responses were also boosted by the re-challenge with a high-dose of the same challenge virus. These data indicate that both low- and high-dose challenges can induce a similar boosting effect.

Our detailed analysis of anti-V3 responses using the actual V3 peptides revealed a significant boosting of immunogen-induced anti-HIV 1084i V3 binding Abs after the multiple low-dose challenges in five out of five vaccinees tested. Importantly, the same boosting was noticed in the two completely protected animals. This is remarkable considering that no viremia was ever detected for >3 years. In addition to these altered binding Ab titers, we also observed an increase in neutralizing activity against the challenge virus in the same aviremic RMs. Peptide absorption linked these increased nAb titers to anti-V3 responses. Interestingly, most of the induced cross-neutralizing anti-V3 Abs targeted the antigenically different version of the V3 crown presented by the challenge virus. Based on these results, we propose that the multiple live-virus exposures boosted anti-HIV-1 responses and altered their specificity in the absence of any detectable viremia.

Pre-existing antiviral immunity has been considered problematic for the recognition of antigenically diverse strains by the “primed” immune system. This idea of a compromised immune system was first discussed in the context of influenza virus antigens [34] and termed Original Antigenic Sin (OAS) [35]. Ab formation during initial influenza infections in childhood was believed to greatly influence future Ab formation against newer strains encountered later [34]. More specifically, vaccination with one strain of influenza virus and subsequent exposure to a heterologous strain would induce anamnestic Ab responses against the initial strain [35]. Later, Nara and coworkers expanded the concept of OAS to the model of deceptive imprinting and defined it as a mechanism leading to a fixed state of immunity which in turn fails to adapt to a changing, but similar pathogen [36,37]. Moreover, they described the induction of clonally restricted B-cell responses due to immunodominant epitopes found on the original antigen (reviewed in [38]). With regard to HIV-1 infections, it was postulated that initial vaccine-induced nAb responses would target immunodominant epitopes of variable gp120 regions (especially the V3 loop), which mutate due to immune selection pressure [36]. Thus, nAb responses would be limited to the primary virus variant, which is considered potentially problematic during chronic HIV-1 infection, as well as for the formation of effective nAb responses against non-homologous HIV-1 strains in vaccine recipients [36-39]. Yet, we [40] and others [41-43] gave evidence that the occurrence of OAS is not absolute.

Investigating the anti-V3 binding Ab repertoire in vaccine-protected RMs, we indeed detected a statistically significant boosting of the initial vaccine-induced binding Abs after live-virus encounters with the heterologous strain, supporting the concept of OAS. However, our data do not imply a limitation in subsequent Ab responses against non-homologous virus strains as suggested by the model of deceptive imprinting [36,37]. Based upon the initial selection of V3 mimotopes and their specific recognition of plasma samples after but not before live SHIV-C exposures, we demonstrate a change in the Ab repertoire as a consequence of live-virus encounters. This neo-antigen reactivity was then confirmed indirectly by peptide absorption analysis showing the induction of nAbs against an antigenically different version of the same V3 epitope. Thus, despite the pre-existing anti-V3 Abs present after immunization, virus-challenged RMs that never developed viremia produced cross-neutralizing anti-V3 Abs targeting the epitope version presented by the challenge virus.

Of note, the boosting of virus-specific Ab responses as a consequence of live-virus exposures that failed to cause detectable viremia was not restricted to Env only. According to our recent work [16], multiple live-virus exposures also affected anti-Tat Ab responses; we showed that mimotopes displaying the N-terminus of HIV-1 Tat were only recognized by Abs of protected vaccinees, RRi-11 and RTr-11, *after* the live-virus exposures (week 7), but not on the day of challenge (week 0). Subsequent quantitative ELISAs using the full-length HIV-1 Tat protein and Tat peptides confirmed an increase of anti-Tat Abs after multiple exposures to SHIV-C in three out of four aviremic vaccinees (RRi-11, RTr-11 and RAt-9). These data indicate that the virus challenges altered Ab responses against at least two different HIV-1 proteins, Env and Tat, in the absence of systemic infection.

Animals ROb-12, RAt-9, RRi-11 and RTr-11 were never viremic. How did the multiple low-dose virus challenges alter the vaccine-induced anti-Env and anti-Tat Ab repertoire in these animals? Theoretically, three mechanisms could be responsible: i) cryptic infection of target cells and their subsequent lysis. This may have produced sufficiently high concentrations of challenge virus proteins to induce Abs with altered specificities; ii) formation of immune complexes of virions and/or soluble protein with pre-existing, vaccine-induced Abs followed by efficient binding to and presentation by Fc-gamma receptor (FcγR)-expressing cells; iii) a combination of the two mechanisms.

In order to estimate potential mechanisms that may have been involved, we first employed an ultrasensitive HIV-1 gp120 antigen capture ELISA and used parental SHIV-1157ip gp120 [30] as reference protein. Total gp120 concentration of the virus stock solubilized in disruption buffer was only 35 pg per challenge virus dose. This total amount of gp120 is about 10^6^-10^7^ less than what has been used for standard immunizations in humans or macaques [44-48], making boosting via soluble Env a highly unlikely mechanism – especially since no adjuvant was involved in contrast to the standard vaccination/boosting protocols [44-48]. Moreover, the virus-induced boosting effect was not only detected in animals from one study [12] but in four animals derived from three different studies ([8,12] and unpublished). Thus, we propose that this observation is not virus-stock dependent since the challenge virus strains varied among the different studies.

Cryptic infection is expected to boost antiviral cellular immunity. This was observed in some of the vaccine-protected RMs (RTr-11, RAt-9, ROb-12). In contrast, vaccinee RRi-11 had no anamnestic cellular immune responses and thus fulfilled the criteria for sterilizing immunity. For this animal, we propose that vaccine-induced nAbs not only blocked initial infection of target cells but also led to the formation of virion-antibody complexes (reviewed in [49]). Such opsonized virions may then have been taken up by follicular dendritic cells in lymph nodes, which in turn resulted in more effective presentation of Env epitopes to B cells (reviewed in [50,51]). Thus, we suggest that virions – although unable to cause systemic infection – might have acted as effective immunogens in the form of antigen-antibody complexes.

Ab-covered virions also altered the anti-Tat Ab responses. According to Monini et al. [52], HIV-1 Tat can form a specific molecular complex with trimeric Env and hence is found on virion Env spikes. This observation would explain the boosting of pre-existing or induction of novel anti-Tat Ab responses.

In the current study, we examined the Env-specific Ab responses in five completely protected vaccinees and chose two of them to investigate the virus-induced consequences on pre-existing Ab responses further. At this point, it should be emphasized that well studied individual cases, as the protected monkeys described above, can shed light onto important basic mechanisms and potential solutions to the problems of prevention and/or cure of retroviral infections. To illustrate the power of case reports, we point to the description of a monkey with breakthrough SIV infection [53], HIV-1 superinfection in a relatively recently infected individual [54], and the “Berlin patient” [55].

In summary, we described a boosting of pre-existing, vaccine-induced Ab responses in immunized macaques that remained aviremic throughout all heterologous SHIV-C challenges. Furthermore, detailed epitope mapping revealed newly induced Abs specific for epitopes formed only in the challenge virus.

## Conclusions

Exposures to live virus that fail to cause viremia can nevertheless result in changes in the Ab repertoire initially induced by vaccination. This is an important finding with implications for the analysis of immunogenicity data for clinical vaccine trials in humans. Sub-threshold virus exposure due to high risk behaviour may broaden the vaccine-induced Ab repertoire in the absence of systemic infection or seroconversion. Repeated live-HIV-1 exposures may complicate the interpretation of “vaccine-only” immune responses, which will be difficult to investigate. Thus, further well-controlled challenge studies in biological relevant primate models will be necessary to elucidate the underlying mechanism(s) of low-dose pathogen interactions with vaccinated hosts without overt infection.

## Methods

### Animals

Indian-origin RMs (*Macaca mulatta*) were housed at the Yerkes Regional Primate Research Center (Atlanta, GA, USA). All procedures were approved by the Animal Care and Use Committees of Emory University and the Dana-Farber Cancer Institute (DFCI). To examine Env-specific Ab responses induced by live-virus exposure, plasma samples were collected from RMs that had been either part of a vaccine/challenge study or an immunogenicity study. Animals of the former included ROb-12 (unpublished), RAt-9 [8-10], RQe-10 [11], RRi-11, RTr-11, RGe-11, RBr-11 and RDo-11 [12]. The immune status of these vaccinees as well as immunogens and challenge viruses used are summarized in Table 1.

Group 1 animals [12] described in Figure 1 had received three protein immunizations, consisting of HIV-1 gp160, HIV-1 Tat, and SIV Gag-Pol particles (weeks− 33, -27 and −2). Starting two weeks after the third protein immunization (week 0), all RMs were given five weekly low-dose intrarectal (i.r.) challenges of SHIV-1157ipEL-p (weeks 0–4, 8,000 50% tissue culture infectious doses (TCID_50_, measured by TZM-bl assay [20]). At week 7, animals without evidence of viremia were rechallenged i.r. with a single high dose of the same virus (1.5 × 10^5^ TCID_50_).

Group 2 animals shown in Figure 1 (RDs-12, RFg-12, RYc-12, RGn-12, RNu-12, RMc-12, RJd-12, RGv-12; unpublished) had been treated according to a similar immunization protocol as the vaccinated and challenged animals (involving HIV-1 1084i gp160, SIV Gag-Pol particles, HIV-1 Tat) with the addition of SIV Nef overlapping synthetic peptides (OSP). All recombinant protein immunogens were given in incomplete Freund’s adjuvant (IFA).

### Protein ELISA

Microtiter plates (Greiner-Bio-One GmbH, Frickenhausen, Germany) were coated with CN54-gp140 (Polymun, Scientific GmbH, Klosterneuburg, Austria); 1 μg/ml in 50 μl/well carbonate-bicarbonate buffer (Sigma-Aldrich, St. Louis, MO, USA). After blocking for 2 h at room temperature with 3% casein (Sigma-Aldrich), plasma samples were diluted 3-fold, added to the plates (in blocking buffer) and incubated for 3 h at room temperature. Afterwards, Ab binding was detected using a HRP-conjugated anti-monkey IgG (1 h at room temperature, 1:2,000 in blocking buffer; Sigma-Aldrich) and o-Phenylenediamine dihydrochloride (OPD; Amresco Inc., Cochran, Solon, OH, USA) + H_2_O_2_. After stopping the reaction with 1 N H_2_SO_4_, plates were read at 490/620 nm. Binding Ab titers were calculated by linear regression and defined as reciprocal plasma dilution with an absorbance 5× higher than the background absorbance, detected with the autologous pre-immune plasma.

Since the five completely protected RMs had been enrolled in four different immunization/challenge studies, the time points of the samples collected varied slightly in Figure 1E. For the “before any live-virus challenge” time point we always used week 0, but in case of RAt-9, we analyzed week −2 because week 0 was not available. For the “after live-virus challenge” samples we analyzed the following time points: ROb-12 plasma from week 6, RAt-9 plasma from week 30, RQe-10, RRi-11 and RTr-11 plasma from weeks 6 or 7, respectively.

### Subtractive biopanning

Plasma samples of vaccinated, protected animals were used to identify specific Ab epitopes by peptide phage-display. Paramagnetic beads (Dynabeads M-280 tosylactivated; Invitrogen, Carlsbad CA, USA) were coated with rabbit anti-monkey IgG (as previously described [26]) and pre-incubated with polyclonal RM plasma (collected at week 7, positive selection). After overnight incubation with the original phage-display libraries (7mer, cyclic 7mer, 12mer; New England Biolabs, Ipswich MA, USA), bound phages were eluted by pH shift with 0.2 M glycine-HCl pH 2.2 supplemented with 1 mg/ml bovine serum albumin (BSA) and neutralized with 1 M Tris–HCl pH 9.1 (Sigma-Aldrich). Eluted phages were used in a negative selection (plasma sample from week 0). Remaining phages were amplified in *Escherichia coli* (ER2738, New England Biolabs), precipitated overnight at 4°C (20% PEG-8000/2.5 M NaCl; Fisher Scientific, Fair Lawn NJ, USA) and subjected to two more rounds of selection. After the third positive selection, phages were titered, single clones were picked and then tested by phage ELISA for specific binding. Single-stranded DNA of positive clones was sequenced. Peptide sequences were grouped into motifs and assigned to the gp160 sequence of the vaccine- (HIV1084i) or challenge strain (SHIV-1157ipEL-p).

### Phage ELISA and cross-reactivity profile of mimotopes

Phage ELISAs were performed as previously published [26]. As a negative control, M13KO7 helper phages (New England Biolabs) without peptide insert were included (wild type phage). Phage peptides mimicking the V3 crown were evaluated according their binding activity to polyclonal plasma Abs of different time points before and after virus challenges. Optical density (OD) signals at least 3× higher than signals detected with the negative control were considered positive and the cross-reactivity profile of each mimotope was expressed as color-coded heat-map.

### Synthetic peptides

Peptides corresponding to the V3 consensus clade C sequence were obtained from the NIH AIDS Reagent and Reference Reagent Program (15 amino acids (AA) long, overlapping by 11 AA). Peptides corresponding to HIV1084i V3 were 15 AA long, overlapping by 10 AA (CHI-Scientific, Maynard, MA, USA) (sequences see Figure 2C). A pool of three overlapping peptides was used (peptide set). As negative control, a scrambled C-terminal gp120 peptide was included (24 AA, GVTKYIPGSIPVEGLKSHKAGSYK, Molecular Biology Core Facilities, DFCI, Boston, MA).

### Peptide ELISA

Microtiter plates were coated with a pool of three overlapping V3 peptides or the negative control peptide (total concentration per peptide set, 2 μg/ml in 100 μl/well carbonate-bicarbonate buffer). After blocking for 2 h at room temperature with 3% casein, plasma samples were diluted 2-fold and added to the plates (in blocking buffer). Ab detection and calculation of binding Ab titers as described for the protein ELISA. Ab titers were compared at weeks 0 and 7.

### Depletion of nAbs using synthetic peptides

Plasma samples were incubated in the presence/absence of V3 peptides (based on [40]) and tested against SHIV-1157ipEL-p and SHIV_SF162P4_ by TZM-bl assay [20]. Briefly, a total of 5,000 cells/well were seeded overnight. Plasma samples were diluted 2-fold and incubated for 1 h at 37°C with a pool of three overlapping peptides (total concentration, 50 μg/ml), the control peptide, or an equal volume of growth medium. Virus was incubated with the Ab/peptide mixture for 1 h at 37°C and afterwards transferred into the 96-well flat-bottom plate containing TZM-bl cells. After maximally 20 h, medium was exchanged. After another 24 h, Bright-Glo luciferase substrate (Promega, Madison, WI, USA) was used to measure luciferase activity. Neutralization titers are calculated with regard to autologous pre-immune plasma samples and expressed as the reciprocal plasma dilution inhibiting 50% of virus infection (IC_50_). The ability of peptides to block nAbs is calculated as the percent reduction in neutralization titers relative to that of the corresponding plasma sample without peptide incubation.

### Detection of Env protein in the challenge virus preparation

100 μl/well of the original virus preparation were used in the HIV-1 gp120 Antigen Capture Assay (Advanced Bioscience Laboratories, Rockville, MD, USA) according to the manufacturer’s instructions. For more accurate estimation of the SHIV-1157ipEL-p gp120 content, we included the parental 1157ip gp120 protein as reference [30].

### Statistical analysis

Statistics were calculated using a paired, two-tailed Student’s t-test comparing the significance between Ab titers before and after five low-dose challenges. Differences with *P* < 0.05 were considered statistically significant. Differences in nAb titers detected with plasma samples in the absence/presence of certain peptides were calculated using an unpaired, two-tailed Student’s t-test (including Bonferroni correction: *P* < 0.025 were considered statistically significant). All statistical analysis was performed using GraphPad Prism 5 for Windows, GraphPad Software.

## Competing interest

The authors declare no competing financial interests.

## Authors’ contributions

BCB, MH and RMR designed the experiments and wrote the manuscript. BCB performed and analyzed the experiments. SKL and RAR performed the immunization studies. All authors read and approved the final manuscript.
